# Supplementary material for: End Sequence Analysis Toolkit (ESAT) expands the extractable information from single-cell RNA-seq data
Source: Genome Res. 2016 Oct;26(10):1397–410. doi: 10.1101/gr.207902.116 (PMC5052061; doi:10.1101/gr.207902.116)
Supplement: Supplemental Material [file supp_gr.207902.116_Supp_Materials.tar.gz › ESAT-esat_v0.1/doc/html/classbroad_1_1core_1_1math_1_1_mann_whitney.html]

ESAT: broad.core.math.MannWhitney Class Reference


ESAT


- Main Page
- Related Pages
- Packages
- Classes
- Files

- Class List
- Class Index
- Class Hierarchy
- Class Members

All Classes Namespaces Files Functions Variables Enumerator Pages

- broad
- core
- math
- MannWhitney

Public Member Functions |
Static Protected Member Functions |
List of all members

broad.core.math.MannWhitney Class Reference

|  |  |
| --- | --- |
| Public Member Functions | |
|  | MannWhitney (double[] sample1measurements, double[] sample2measurements) |
|  | |
|  | MannWhitney (ArrayList< Double > sample1measurements, ArrayList< Double > sample2measurements) |
|  | |
| double | getZ () |
|  | |
| double | getPvalue () |
|  | |

|  |  |
| --- | --- |
| Static Protected Member Functions | |
| static List< Double >[] | rankOrder (TreeMap< Double, List< String >> measurements) |
|  | |
| static double | rank (double count, List< String > list) |
|  | |

## Detailed Description

Author
:   prussell Mann Whitney U test

## Constructor & Destructor Documentation

|  |  |  |  |
| --- | --- | --- | --- |
| broad.core.math.MannWhitney.MannWhitney | ( | double[] | *sample1measurements*, |
|  |  | double[] | *sample2measurements* |
|  | ) |  |  |

Instantiate with arrays of measurements

Parameters
:   |  |  |
    | --- | --- |
    | sample1measurements | Sample 1 measurements |
    | sample2measurements | Sample 2 measurements |

Here is the call graph for this function:

|  |  |  |  |
| --- | --- | --- | --- |
| broad.core.math.MannWhitney.MannWhitney | ( | ArrayList< Double > | *sample1measurements*, |
|  |  | ArrayList< Double > | *sample2measurements* |
|  | ) |  |  |

Instantiate with lists of measurements

Parameters
:   |  |  |
    | --- | --- |
    | sample1measurements | Sample 1 measurements |
    | sample2measurements | Sample 2 measurements |

Here is the call graph for this function:

## Member Function Documentation

|  |  |  |  |  |
| --- | --- | --- | --- | --- |
| double broad.core.math.MannWhitney.getPvalue | ( |  | ) |  |

Get P value

Returns
:   P value

|  |  |  |  |  |
| --- | --- | --- | --- | --- |
| double broad.core.math.MannWhitney.getZ | ( |  | ) |  |

Get the test statistic

Returns
:   The test statistic

|  |  |  |  |
| --- | --- | --- | --- |
| static double broad.core.math.MannWhitney.rank | ( | double | *count*, |
|  |  | List< String > | *list* |
|  | ) |  |  |

staticprotected

Here is the caller graph for this function:

|  |  |  |  |  |  |
| --- | --- | --- | --- | --- | --- |
| static List<Double> [] broad.core.math.MannWhitney.rankOrder | ( | TreeMap< Double, List< String >> | *measurements* | ) |  |

staticprotected

Get the overall ranks of measurements from both samples 1 = low, N = high

Parameters
:   |  |  |
    | --- | --- |
    | measurements | Map of each measurement to the set of samples it belongs to (one or both samples) |

Returns
:   List whose first element is the list of sample 1 ranks and whose second element is the list of sample 2 ranks

Here is the call graph for this function:

Here is the caller graph for this function:

---

The documentation for this class was generated from the following file:

- java/broad/core/math/MannWhitney.java


---

Generated on Fri Aug 15 2014 15:10:53 for ESAT by  

 1.8.7
